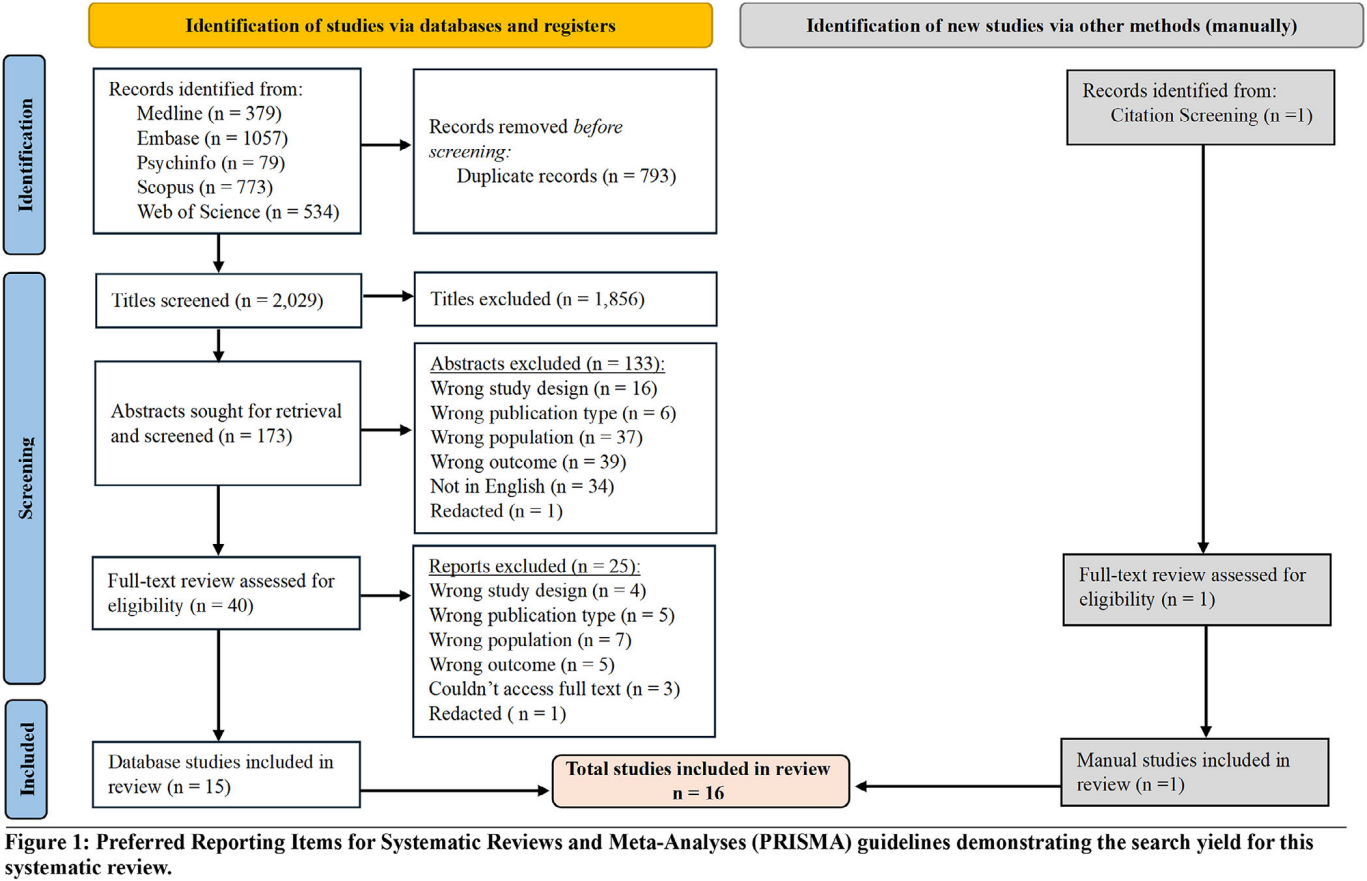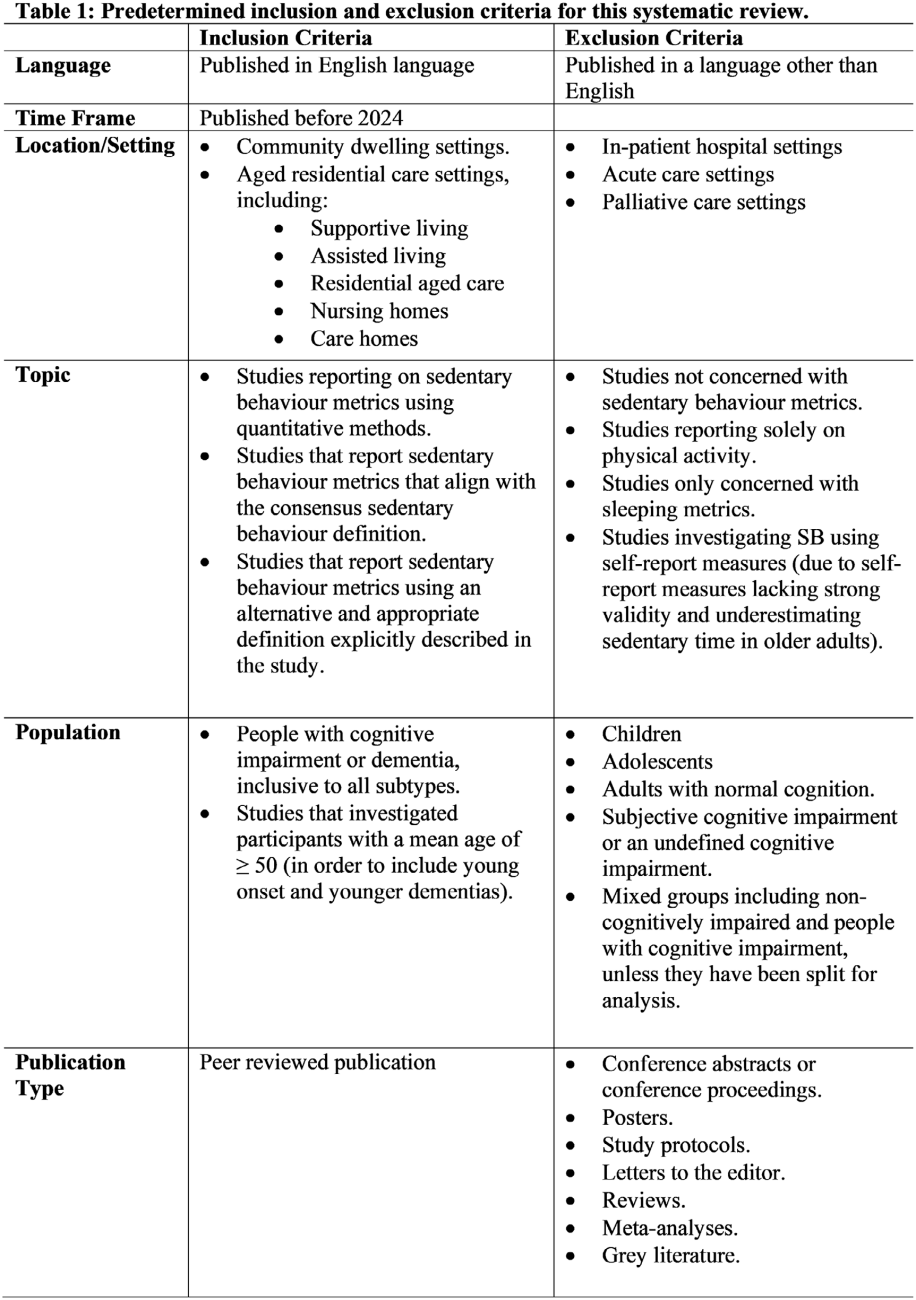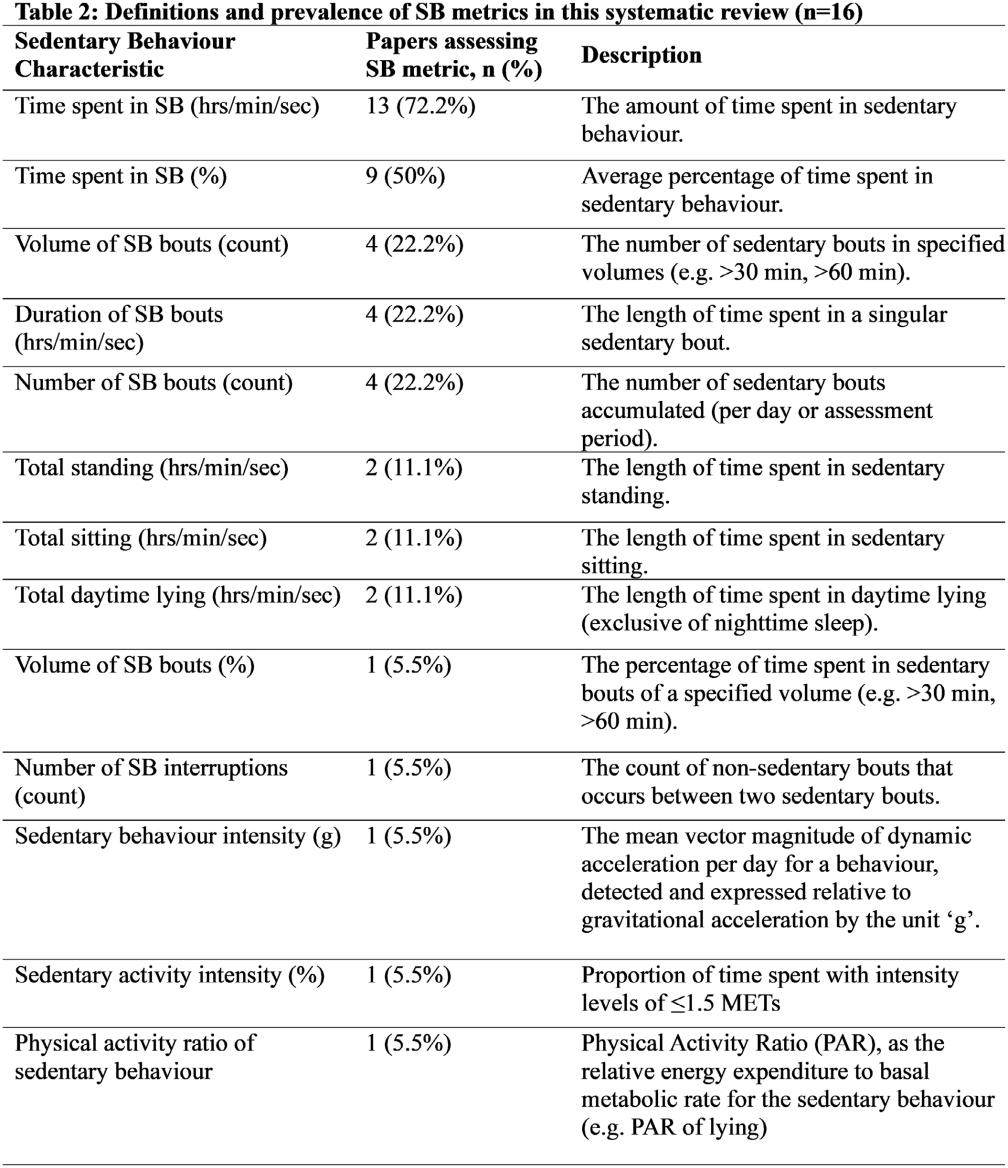# Identifying the methods and metrics used to assess and describe sedentary behaviour in people with cognitive impairment: A systematic review

**DOI:** 10.1002/alz70858_099653

**Published:** 2025-12-25

**Authors:** Jenny L Wales, Chloe Hinchliffe, Ryan S Falck, Alison J Yarnall, Silvia Del Din, Ríona Mc Ardle

**Affiliations:** ^1^ National Institute for Health and Care Research, Biomedical Research Centre, Newcastle upon Tyne, Tyne and Wear, United Kingdom; ^2^ Translational and Clinical Research Institute, Newcastle University, Newcastle upon Tyne, Tyne and Wear, United Kingdom; ^3^ University of British Columbia, Vancouver, BC, Canada; ^4^ NIHR Newcastle Biomedical Research Centre, Newcastle University, Newcastle upon Tyne, Tyne and Wear, United Kingdom

## Abstract

**Background:**

Greater sedentary behaviour (SB) is associated with adverse outcomes, including loss of functional independence, disability, and dementia. Few studies have investigated SB in people living with cognitive impairment (PwCI), including mild cognitive impairment (MCI) and dementia. Current evidence suggests PwCI are more sedentary than their peers without cognitive impairment, highlighting the need to reduce SB to improve health and mitigate disability. However, our understanding of how to accurately and reliably measure SB in PwCI is limited and precludes developing effective methods to reduce SB. Device‐based measures provide sensitive estimates of SB, although it is unclear whether these devices are accurate and reliable across PwCI. The lack of synthesised literature limits understanding of the common measures used to investigate SB in PwCI. To address knowledge gaps, we conducted a systematic review to identify the current device‐based methods for assessing SB in PwCI.

**Methods:**

This systematic review was conducted following Preferred Reporting Items for Systematic Reviews and Meta‐Analyses (PRISMA) guidelines (Figure 1). 2,029 articles were identified following initial search, 16 were included based on predetermined criteria (Table 1) following a review of titles, abstracts and full texts.

**Results:**

Of the included 16 studies (total *N* = 1,675 (range: 8‐414), 55% female, age range: 68‐91.7) seven studies investigated MCI, seven investigated dementia and two investigated both MCI and dementia. Cognitive impairment was assessed via the Mini Mental State Examination (MMSE) (*n* = 11, score range:15.5‐28.65), and the Montreal Cognitive Assessment (MoCA) (*n* = 5, score range:13.1‐24) and the Clinical Dementia Rating Scale (CDR) (*n* = 1). All studies employed accelerometery to measure SB in PwCI. Ten different accelerometer devices were used; devices were placed on the wrist (*n* = 7), trunk (*n* = 8), and thigh (*n* = 1). Metrics used to describe SB were commonly related to SB volume (Table 2).

**Conclusion:**

The results of this review will guide future selection of digital tools and measures to investigate SB in PwCI. Recommendations will advise the most appropriate protocols to monitor and measure SB in PwCI, supporting personalised care across cognitive impairment and care settings.